# The AEROPATH project targeting *Pseudomonas aeruginosa*: crystallographic studies for assessment of potential targets in early-stage drug discovery

**DOI:** 10.1107/S1744309112044739

**Published:** 2012-12-25

**Authors:** Lucille Moynie, Robert Schnell, Stephen A. McMahon, Tatyana Sandalova, Wassila Abdelli Boulkerou, Jason W. Schmidberger, Magnus Alphey, Cyprian Cukier, Fraser Duthie, Jolanta Kopec, Huanting Liu, Agata Jacewicz, William N. Hunter, James H. Naismith, Gunter Schneider

**Affiliations:** a Biomedical Sciences Research Complex, University of St Andrews, St Andrews KY16 9ST, Scotland; b Department of Medical Biochemistry and Biophysics, Karolinska Institutet, S-171 77 Stockholm, Sweden; c Division of Biological Chemistry and Drug Discovery, College of Life Sciences, University of Dundee, Dundee DD1 5EH, Scotland

**Keywords:** protein structure, Gram-negative bacteria, *Pseudomonas aeruginosa*, infectious diseases, structure-based inhibitor design

## Abstract

Bacterial infections are increasingly difficult to treat owing to the spread of antibiotic resistance. A major concern is Gram-negative bacteria, for which the discovery of new antimicrobial drugs has been particularly scarce. In an effort to accelerate early steps in drug discovery, the EU-funded AEROPATH project aims to identify novel targets in the opportunistic pathogen *Pseudomonas aeruginosa* by applying a multidisciplinary approach encompassing target validation, structural characterization, assay development and hit identification from small-molecule libraries. Here, the strategies used for target selection are described and progress in protein production and structure analysis is reported. Of the 102 selected targets, 84 could be produced in soluble form and the *de novo* structures of 39 proteins have been determined. The crystal structures of eight of these targets, ranging from hypothetical unknown proteins to metabolic enzymes from different functional classes (PA1645, PA1648, PA2169, PA3770, PA4098, PA4485, PA4992 and PA5259), are reported here. The structural information is expected to provide a firm basis for the improvement of hit compounds identified from fragment-based and high-throughput screening campaigns.

## Abstract 2

A focused strategy has been directed towards the structural characterization of selected proteins from the bacterial pathogen *P. aeruginosa*. The objective is to exploit the resulting structural data, in combination with ligand-binding studies, and to assess the potential of these proteins for early-stage antimicrobial drug discovery.

## 1. Introduction

*Pseudomonas aeruginosa* is a Gram-negative pathogen responsible for a significant level of hospital-acquired infections, particularly in burns victims and immunocompromised and cystic fibrosis patients (Kerr & Snelling, 2009 ▶; Ratjen & Döring, 2003 ▶). Two major factors contribute to the success of this opportunistic pathogen. Firstly, *P. aeruginosa* is able to survive in moist environments with low nutrient supply and can establish itself in niches characteristic to the clinical environment. Secondly, the bacterium is highly adapted to acquire antibiotic resistance and many strains have been identified that employ common mechanisms such as modification of the drug or its target, active efflux and/or decreased uptake of drugs (Breidenstein *et al.*, 2011 ▶; Livermore, 2002 ▶). The need for novel and improved antibiotics to tackle *P. aeruginosa* and related drug-resistant Gram-negative bacteria has been well recognized, along with the practical difficulties associated with antibacterial drug development (Payne *et al.*, 2007 ▶; Shlaes, 2003 ▶).

Armed with annotated genome sequences from important Gram-negative pathogens, increasing knowledge of the mechanism of action of existing drugs and some data on gene essentiality, we have pursued potential drug targets in *P. aeruginosa*. In support of our study, we are assisted by an improved understanding of the types of molecules that are likely to provide either drug targets (Hunter, 2009 ▶) or appropriate lead compounds (O’Shea & Moser, 2008 ▶).

A thorough assessment of a potential drug target requires an efficient source of pure material for structural and ligand-binding studies, an accurate crystallographic model, compound-screening data using fragment or designed libraries and, where possible, structure–activity relationships for groups of inhibitors. This information would also underpin the search for new inhibitors that might represent useful lead compounds. A multidisciplinary approach has been implemented towards these objectives in the AEROPATH project (http://www.aeropath.eu/). A bio-chemoinformatics analysis of the *P. aeruginosa* genome and of published information on ligands for the targets was achieved in the course of the study (G. J. Bickerton, I. M. Carruthers, W. N. Hunter & A. L. Hopkins, in preparation). Screening of fragment libraries and a collection of bioactive molecules has been carried out using differential scanning fluorimetry (DSF) and nuclear magnetic resonance (NMR) spectrometry. Appropriate enzyme assays have been developed and then applied in high-throughput screens (HTS; Eadsforth *et al.*, 2012 ▶). A subset of targets has been subjected to *in vivo* studies to elucidate whether single-gene-knockout strains of *P. aeruginosa* strain PAO1 (Schnell *et al.*, 2012 ▶) are capable of establishing infection in the lungs of mice. However, at the core of the project is the derivation of structural models of the potential targets. The crystal structures allow a druggability analysis of the active sites (Krasowski *et al.*, 2011 ▶) and are essential to support the derivation of the structure–activity relationships of the ligands identified.

Here, we describe the strategies chosen for target selection, protein production and a medium-throughput approach to derive three-dimensional information for the selected *P. aeruginosa* proteins. Of the 102 targets, 84 could be produced in soluble form and 37 *de novo* crystal structures and two NMR structures of these proteins have been obtained. We report the crystal structures of a subset of these targets ranging from well characterized metabolic enzymes to proteins with unknown functions.

## 2. Methods

### 2.1. Target selection and construct design

Target selection was based on the available genome sequence and preliminary annotation of *P. aeruginosa* strain PAO1 (Stover *et al.*, 2000 ▶) together with results of mutagenesis studies to identify potentially essential genes (Jacobs *et al.*, 2003 ▶; Liberati *et al.*, 2006 ▶). Target selection also involved other considerations such as feasibility of enzyme assay, chemogenomics information and an appropriate balance of novel uncharacterized proteins *versus* established targets for antibacterial drug design. In a few cases we selected close orthologues if the target from PAO1 proved intractable. For particular targets, the genes from *Actinobacter baumannii* (Fyfe *et al.*, 2009 ▶), *Burkholderia cenocepacia* (Morgan *et al.*, 2011 ▶) and *Serratia marcesens* (Rao *et al.*, 2011 ▶) have proven useful.

The coding sequences of the chosen genes of the reference strain PAO1 were obtained from the website of the *Pseudomonas* genome annotation database (http://www.pseudomonas.com). Signal sequences (Petersen *et al.*, 2011 ▶) were removed, but otherwise full-length proteins were initially cloned. In a small number of cases, further constructs were made to overcome issues with insoluble expression. The coding sequences were amplified by PCR using *PfuTurbo* polymerase (Stratagene), LongTemplate polymerase (Roche) or Long PCR (Fermentas) for GC-rich sequences. Targets were cloned into one of five vectors, each providing an N-terminal cleavable His_6_ tag: pET28a (thrombin-cleavage site), pNIC28-Bsa4 [tobacco etch virus (TEV) cleavage site], a modified Gateway pDEST vector (TEV cleavage site; Oke *et al.*, 2010 ▶), pEHISTEV and pEHISGFPTEV (TEV cleavage site; Liu & Naismith, 2009 ▶) (depending on convenience and laboratory practice at the time). When the modified pDEST vector was employed, the common oligonucleotide 5′-GGGG**ACAAGTTTGTACAAAAAAGCAGGC** **T**TCG**AAGGAG**ATATA**CATATG**TCGTACTACCATCACCATCACCATCACGATTACGATATCCCAACGACC**GAAAACCTGTAT** **TTTCAGGGC**-3′ was used in PCR alongside two gene-specific oligonucleotides. In two cases (PA0254 and PA1165), where PCR amplification failed (possibly owing to a high GC content of 72.6%), synthetic codon-optimized versions were sourced (GenScript). All sequences of the cloned genes were confirmed by DNA sequencing (Eurofins MWG Operon, Ebersberg, Germany).

### 2.2. Expression screening, scale up and purification

Expression screening was carried out in the *Escherichia coli* host strains BL21(DE3) and C43(DE3) at 310 and 294 K in 2 ml LB cultures. The cultures were induced at mid-log phase (OD_600_ = 0.4–0.6) and expression was induced by the addition of isopropyl β-d-1-thiogalactopyranoside to 0.1 m*M* concentration. After incubation, the cells were harvested by centrifugation and broken up using the BugBuster reagent (Novagen) or by sonication supplemented with DNase I (2 µg ml^−1^) in a total volume of 200 µl. The lysate was cleared by centrifugation and filtration through a 0.2 µm pore-size Eppendorf tube-adapted filter (Amicon). SDS–PAGE was used to analyse the total, soluble and insoluble protein distribution of lysed cells. In addition, the fraction which could be bound to 10 µl Ni^2+^–NTA beads (Qiagen) and eluted with 300 m*M* imidazole was examined. Proteins which gave soluble material were scaled up (typically to 1.5–4 l cultures depending on the level of expression in the small-scale experiments) in LB medium supplemented with the appropriate antibiotic. Temperature, growth time, antibiotic and induction conditions followed the small-scale experiments. Cell pellets were thawed and resuspended in 10 m*M* Tris–HCl pH 8.0, 300 m*M* NaCl, 10 m*M* imidazole or 10 m*M* sodium phosphate pH 7.4, 500 m*M* NaCl, 10 m*M* imidazole, 10% glycerol. Lysozyme (Sigma), DNase I (Roche) and EDTA-free protease-inhibitor cocktail tablets (Roche) were added to 0.04 mg ml^−1^, 0.004 mg ml^−1^ and one tablet per 50 ml of lysis buffer, respectively. Cells were lysed either by sonication or by passage through a One Shot cell disruptor (Constant Systems) and were fractionated by centrifugation at 18 000*g* for 25 min to separate the soluble and insoluble fractions. Selenomethionine-substituted proteins were produced according to the metabolic inhibition method (Van Duyne *et al.*, 1993 ▶), while ^13^C- and ^15^N-labelled proteins for NMR structure determination were produced in Spectra 9 medium (Cambridge Isotope Laboratories Inc.). The lysates were applied to immobilized metal-ion chromatography medium (Qiagen and GE Healthcare) in batch mode for 60 min at 277 K with constant agitation. Proteins were eluted with an imidazole gradient and after dialysis were cleaved with either thrombin or TEV protease at 293 K followed by a second Ni^2+^–NTA affinity step to remove uncleaved and contaminating proteins. The final step of the purification protocol involved size-exclusion chromatography using S200 or S75 columns, depending on protein size. Purified proteins were characterized by SDS–PAGE, dynamic light scattering, differential scanning fluorimetry (DSF), mass spectrometry and, where available, enzyme assays.

### 2.3. Protein structure determination

Crystallization screening was performed using Phoenix, Mosquito or Cartesian Honeybee crystallization robots by sitting-drop vapour diffusion. Appropriate protein concentrations were determined using a pre-crystallization test. In the initial screen, the commercial screens JCSG+ and PACT (Qiagen), SaltRx (Hampton Research) and Wizard I and II (Emerald BioSystems) were applied alongside in-house stochastically designed screens (Oke *et al.*, 2011 ▶). Where applicable, cofactors and potential ligands were added to the protein samples. For proteins which were amenable to DSF, screens to identify potential ligands (Ericsson *et al.*, 2006 ▶) or a buffer screen to identify stabilizing conditions prior to the crystallization experiment were performed. Crystal quality was optimized by systematically varying the crystallization conditions.

X-ray diffraction data were collected on beamlines I02, I03 and I04 at Diamond Light Source (Didcot, England), I911-2 and I911-3 at MAX IV Laboratory (Lund, Sweden), and ID23-1, ID14-1, ID14-4, ID29 and BM14 at the European Synchrotron Radiation Facility (Grenoble, France) or in-house using a Rigaku MicroMax-007 HF Cu anode with VariMax optics alongside a Rigaku Saturn 944+ CCD. X-­ray data were processed with *MOSFLM* (Leslie, 2006 ▶), *XDS* (Kabsch, 2010 ▶), *HKL*-2000 (Otwinowski & Minor, 1997 ▶) or in an automated manner with *xia*2 (Winter, 2010 ▶) and were scaled using the *CCP*4 suite (Winn *et al.*, 2011 ▶). The majority of the crystal structures were solved by molecular replacement using the programs *Phaser* (McCoy *et al.*, 2007 ▶) or *MOLREP* (Vagin & Teplyakov, 2010 ▶). Where molecular replacement failed or was not suitable, experimental phasing using sulfur-single wavelength anomalous diffraction (S-­SAD), selenomethionine multi-wavelength and single-wavelength anomalous diffraction (Se-MAD and Se-SAD) or multiple and single isomorphous replacement using heavy-metal derivatives were employed. The locations of heavy and anomalous scattering atoms and initial phases were determined by *SHELXC*/*D*/*E* (Sheldrick, 2008 ▶) and improved with automated model building using *PHENIX* (Adams *et al.*, 2002 ▶) and *CCP*4 (Winn *et al.*, 2011 ▶). Electron-density and difference density maps were inspected and models were adjusted and ligands and solvent molecules were added using *Coot* (Emsley *et al.*, 2010 ▶); they were refined with *REFMAC*5 (Murshudov *et al.*, 2011 ▶) and validated with *MolProbity* (Chen *et al.*, 2010 ▶) and *STAN* (http://xray.bmc.uu.se/usf/www.html).

Analyses of quaternary structures were carried out using the *PISA* server (Krissinel & Henrick, 2007 ▶) and by inspection in *Coot*. Structural comparisons were based on the *SSM* algorithm (Krissinel & Henrick, 2004 ▶) implemented in *Coot* (Emsley *et al.*, 2010 ▶). Searches of the Protein Data Bank for related structures were performed using *DALI* (Holm & Rosenström, 2010 ▶).

## 3. Results and discussion

### 3.1. Target selection

The selected targets (Supplementary Table S1^1^) include several hypothetical proteins, but are primarily proteins involved in metabolic processes known to be important for bacterial survival, growth or virulence. For example, enzymes involved in folate metabolism (Lucock, 2000 ▶), the biosynthesis of fatty acids (Parsons & Rock, 2011 ▶), isoprenoids (Hunter, 2007 ▶), cell-wall components (Vollmer & Seligman, 2010 ▶), lipid A (King *et al.*, 2009 ▶) and rhamnose (Giraud & Naismith, 2000 ▶) or components of the machinery implicated in secretion of virulence factors (Jani & Cotter, 2010 ▶) were selected. A database was established with external links (G. J. Bickerton, I. M. Carruthers, W. N. Hunter & A. L. Hopkins, in preparation) to assist project coordination and to capture information of direct use in the experiments.

### 3.2. Soluble protein into crystallization trials

Most of the cloned genes resulted in soluble proteins in sufficient amounts for crystallization screens using a single-construct approach. In several cases, however, more elaborate screening was required, for instance by the use of several constructs incorporating fused proteins such as glutathione-*S*-transferase or co-expression of chaperones alongside codon-optimized cell lines for protein expression. Of the 84 proteins that were identified as soluble in the small-scale expression screens, we purified 79 on a large scale. Of the five proteins that did not progress, three were owing to failure to scale up and two were abandoned owing to changes in priority. The success rate from the target selection to the crystallization step was 77%, which compares well with those obtained in similar projects using bacterial proteins (Oke *et al.*, 2010 ▶; Lesley *et al.*, 2002 ▶). The crystallization-screening campaigns and subsequent optimization steps resulted in crystals for 47 of the protein samples (Table 1 ▶).

### 3.3. Structure determination

Overall, the project has so far provided *de novo* structural information for 39 proteins: 37 by X-ray crystallography and two by NMR. Of the crystal structures, 24 were determined by molecular replacement and 11 using experimental phasing (Table 2 ▶). In addition to these structures, more than 60 complexes with ligands (cofactors, substrates, inhibitors) have been determined. The crystal structures of some of these targets have already been published by consortium members, in combination with cofactor or ligand complexes (Sainsbury *et al.*, 2011 ▶; Kopec *et al.*, 2011 ▶; O’Rourke *et al.*, 2011 ▶; Schnell *et al.*, 2012 ▶) and the effects of gene knockouts of *P. aeruginosa* in a mouse-infection model (Schnell *et al.*, 2012 ▶). In the following sections, we report several representative target-protein structures ranging from known enzymes to hypothetical proteins of unknown function.

### 3.4. Crystal structure of the uroporphyrinogen III synthase HemD (PA5259)

Uroporphyrinogen III (U3) is the first cyclic tetrapyrrole compound in the haem-biosynthesis pathway and is located at a metabolic branching point (Panek & O’Brian, 2002 ▶). From U3, biosynthetic routes lead to haem, sirohaem or cobalamin (vitamin B_12_). The structure of *P. aeruginosa* uroporphyrinogen III synthase (HemD, PA5259) was solved to 2.4 Å resolution using Se-SAD phasing.

#### 3.4.1. Experimental

The gene coding for HemD was cloned into pET28a (Novagen) with upstream *Nde*I and downstream *Hin*dIII sites (Supplementary Table S2), expressed in *E. coli* BL21(DE3) and purified following the procedures outlined in §[Sec sec2]2. The construct used for structure analysis consisted of full-length HemD (residues 1–251) with three additional residues (Gly-Ser-His) at the N-terminus remaining after tag removal by thrombin cleavage. Crystals of PA5259 were produced by hanging-drop vapour diffusion by mixing 2 µl protein solution at a concentration of 15–36 mg ml^−1^ and 2 µl well solution (0.1 *M* sodium cacodylate pH 6.7, 0.87 *M* sodium citrate). The drops were equilibrated against 1.0 ml reservoir solution. Single crystals were produced by seeding. Crystals of selenomethionine-substituted protein were obtained using a condition identical to that for the native enzyme. The crystals were cryoprotected by transfer into a well solution containing 30%(*v*/*v*) MPD prior to flash-cooling in liquid nitrogen.

Data for the native crystals were collected on beamline ID23-1 and data for SeMet-substituted crystals were collected on beamline ID14-4 at the ESRF (Supplementary Table S3). Data were processed with *MOSFLM* (Leslie, 2006 ▶) and scaled with *SCALA* (Winn *et al.*, 2011 ▶). The HemD crystals belonged to the tetragonal space group *P*4_3_2_1_2 and details of the data statistics are given in Supplementary Table S3. The structure was determined using the SAD protocol of *Auto-Rickshaw* (Panjikar *et al.*, 2009 ▶) and was refined with *REFMAC*5 (Murshudov *et al.*, 2011 ▶). Details of the refinement statistics are given in Supplementary Table S3. The crystallographic data have been deposited in the PDB with accession code 4es6.

#### 3.4.2. Overall structure

**Figure 1:**
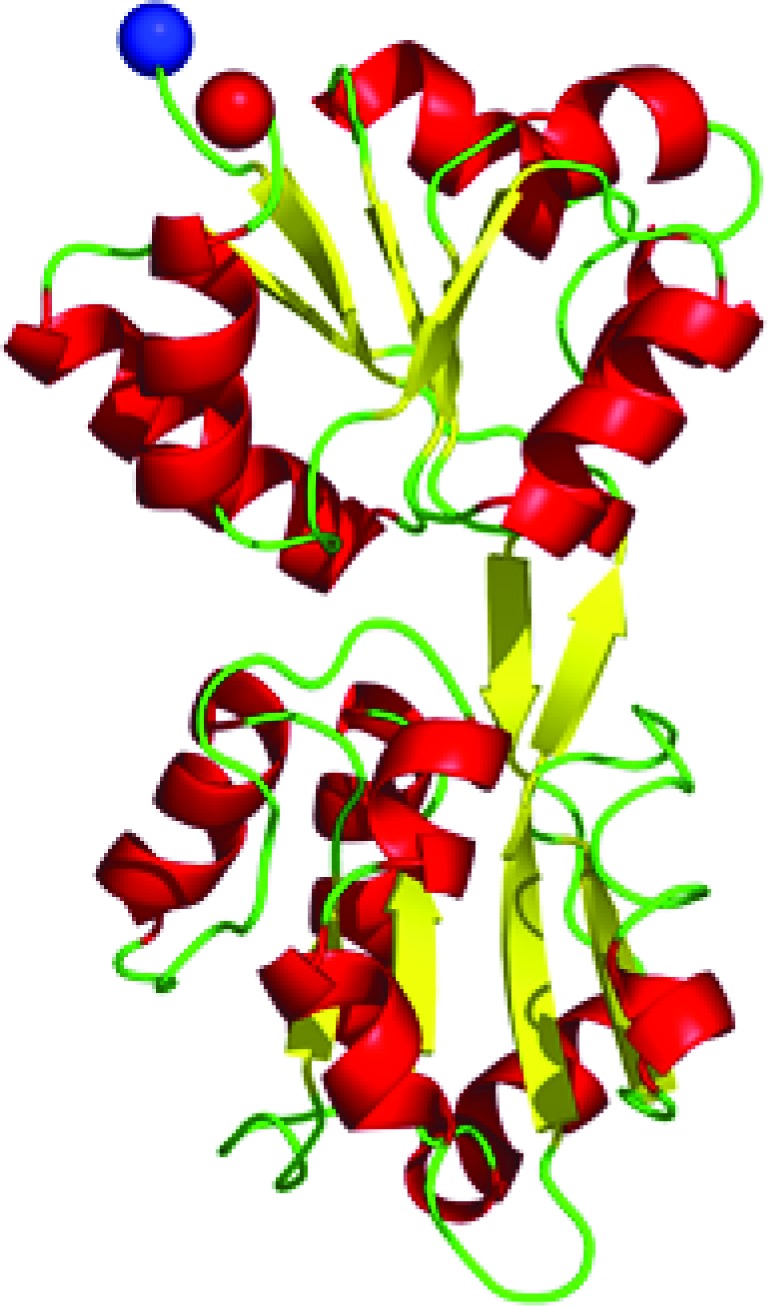
Schematic view of the structure of HemD (PA5259)*.* Secondary-structural elements are colour-coded in yellow (β-strands) and red (α-helices). The N- and C-termini are shown as blue and red spheres, respectively.

*P. aeruginosa* HemD is a monomeric enzyme comprising two globular α/β-domains linked by a pair of antiparallel β-strands (Fig. 1 ▶ and Supplementary Fig. S1*a*). The cavity at the domain interface is sufficiently large to provide space for binding of the linear tetrapyrrole substrate hydroxymethylbilane. The overall fold is identical to that of other known uroporphyrinogen III synthases, but the orientation of the domains is quite different to some of these enzymes (PDB entries 1jr2, 3re1, 3d8n, 3d8r and 1wcx; Mathews *et al.*, 2001 ▶; Schubert *et al.*, 2008 ▶; Peng *et al.*, 2011 ▶; E. Mizohata, T. Matsuura, K. Murayama, H. Sakai, T. Terada, M. Shirouzu, S. Kuramitsu & S. Yokoyama, unpublished work), as reflected by the significant variation in r.m.s.d. values from 1.9 to 5.2 Å upon superposition of the crystal structures. The conformation of PA5259 in the crystals corresponds to the ligand-free closed state of the enzyme (Schubert *et al.*, 2008 ▶). The low level of conservation of active-site residues when compared with the human enzyme (Mathews *et al.*, 2001 ▶) suggests that the development of selective inhibitors of PA5259 might be feasible.

### 3.5. Crystal structure of the unknown protein PA2169

PA2169, annotated as a hypothetical protein, is a representative of the DUF2383 domain of unknown function, with no structure linked to this entry in Pfam (Punta *et al.*, 2012 ▶). Sequence alignments indicated low sequence identity (maximum of 16%) to ferritin-like proteins and domains.

#### 3.5.1. Experimental

The gene coding for PA2169 was cloned into pET28a (Novagen) with upstream *Nde*I and downstream *Hin*dIII sites (Supplementary Table S2), expressed in *E. coli* BL21(DE3) and purified following the procedures outlined in §[Sec sec2]2. The construct used for structure analysis consisted of full-length PA2169 (residues 1–150) with three additional residues (Gly-Ser-His) at the N-terminus remaining after tag removal by thrombin cleavage. Crystallization was performed using the vapour-diffusion method in hanging-drop format by mixing 2 µl protein solution at 20 mg ml^−1^ concentration with 1 µl well solution consisting of 19%(*w*/*v*) PEG 10K, sodium acetate pH 4.6, 20 m*M* strontium chloride. The drops were equilibrated against 1.0 ml well solution. The crystals used for X-ray data collection were cryoprotected with well solution containing 25%(*v*/*v*) ethylene glycol. Crystals in space groups *P*2_1_ and *P*2_1_2_1_2_1_ were obtained under the same conditions. The monoclinic crystals gave better diffraction statistics and therefore structure analysis was pursued using these data. X-ray data were collected on beamline ID23-1 at ESRF. Data were processed with *MOSFLM* (Leslie, 2006 ▶) and scaled with *SCALA* (Winn *et al.*, 2011 ▶). Details of the data statistics are given in Supplementary Table S4. A search model derived from a ferritin-like domain of an uncharacterized protein from *Anabaena variabilis* (PDB entry 3fse; Joint Center for Structural Genomics, unpublished work) was successfully used to solve the structure by molecular replacement, despite a low sequence identity of only 16%. Two polypeptides constitute the asymmetric unit.

The structure was refined with *REFMAC*5 (Murshudov *et al.*, 2011 ▶) and details of the refinement statistics are given in Supplementary Table S4. The crystallographic data have been deposited in the PDB with accession code 4etr.

#### 3.5.2. Overall structure

**Figure 2:**
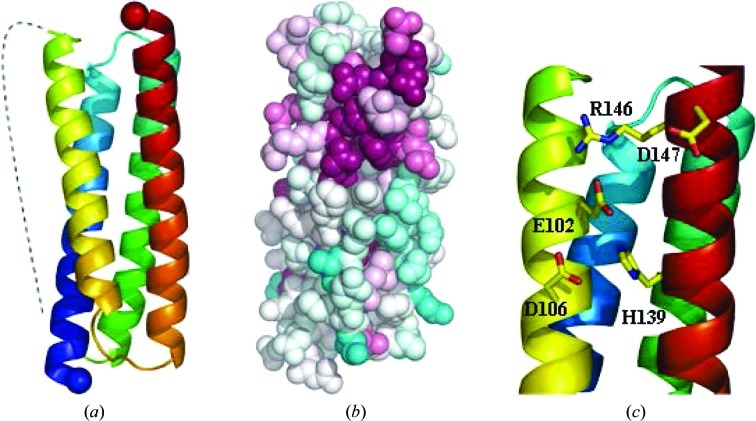
(*a*) Schematic view of the structure of PA2169, a protein of unknown function. The dotted line indicates the flexible loop that is not well defined in electron density. (*b*) Surface representation of PA2169 with residues colour-coded according to sequence conservation from white (not conserved) to cyan (invariant). (*c*) View of the potential metal-binding site in PA2169 comprising residues Glu102, Asp106 and His139.

The structure of PA2169 revealed a four-helix-bundle fold (Fig. 2 ▶ *a* and Supplementary Fig. S1*b*) with the same topology as observed in the ferritin-like module of the redox-defence protein from *Mycobacterium smegmatis* (Roy *et al.*, 2007 ▶). The crystal structure is thus consistent with circular-dichroism spectroscopy using purified protein samples, which suggested an all-α fold for PA2169. A disulfide bond is formed between Cys30 and Cys101. The protein lacks the metal-ion-binding site that is typical of the ferritin family (Theil, 2011 ▶) and the iron-binding residues are not conserved. In the structure of PA2169 a triad composed of Glu102, Asp106 and His139 is found in a different location to the iron-binding site in the ferritin family and resembles a potential metal-binding site. These residues are conserved in proteins belonging to the DUF2383 domain sequence family (Figs. 2 ▶ *b* and 2 ▶ *c*). However, attempts to provide experimental evidence for metal binding using differential scanning fluorimetry and cocrystallization were unsuccessful.

### 3.6. Crystal structure of PA4992, a putative aldo–keto reductase

PA4992 is annotated as a member of the aldo–keto reductase superfamily. These enzymes catalyze the reversible reduction of ketones to the respective alcohols using NAD(P)H as a hydride donor (Jez & Penning, 2001 ▶; Ellis, 2002 ▶).

#### 3.6.1. Experimental

The gene coding for PA4992 was cloned into the pNIC28Bsa4 vector (GenBank accession No. EF198106) using ligation-independent cloning (Supplementary Table S2), expressed in *E. coli* BL21(DE3) and purified following the procedures outlined in §[Sec sec2]2. As removal of the affinity tag by TEV protease resulted in protein precipitation, the uncleaved construct was used for structure analysis, consisting of residues 1–270 (full-length PA4992) and the affinity tag, including a linker at the N-terminus (MHHHHHHSSGVDLGTE­NLYFQS). Rod-shaped crystals of apo PA4992 were grown at 293 K from droplets consisting of 2 µl reservoir solution [0.1 *M* bis-tris methane pH 6.35, 0.1 *M* sodium malonate, 16%(*w*/*v*) PEG 3350] and 2 µl PA4992 solution (12 mg ml^−1^ in 20 m*M* Tris–HCl pH 8.0, 150 m*M* NaCl). The droplets were equilibrated against 1.0 ml reservoir solution. Crystals of the holoenzyme were obtained by incubating the protein with 10 m*M* NADP^+^ at room temperature for 10 min before crystallization. The best crystals of the NADP^+^ complex were grown using 25.5% polyacrylic acid, 0.1 *M* HEPES pH 7.4 as a reservoir solution. Crystals were briefly soaked in well solution containing 25%(*v*/*v*) glycerol before cooling in liquid nitrogen. X-ray data were collected from a crystal of the apoenzyme on beamline I9-11 at MAX IV Laboratory and from a crystal of the holoenzyme on beamline ID21-1 at the ESRF (Supplementary Table S5). Data were processed with *MOSFLM* (Leslie, 2006 ▶) and scaled with *SCALA* (Winn *et al.*, 2011 ▶).

The structure of the holoenzyme was determined by molecular replacement using the coordinates of AKR11C1 from *Bacillus halodurans* (PDB entry 1ynp; Marquardt *et al.*, 2005 ▶) as a template and were refined with *REFMAC*5 (Murshudov *et al.*, 2011 ▶). The final model of holo PA4992 was used to determine the structure of apo PA4992, which was subsequently refined following the same protocol (Supplementary Table S5). At the N-terminus, residues from the tag were defined in the electron density and were included in the model. However, no or weak electron density was present for three flexible loop regions, 33–41, 180–181 and 222–226, and the last two C-terminal residues, indicating disorder, and these residues were therefore not modelled. The crystallographic data have been deposited in the PDB with accession codes 4exb (apoenzyme) and 4exa (holoenzyme).

#### 3.6.2. Overall structure

**Figure 3:**
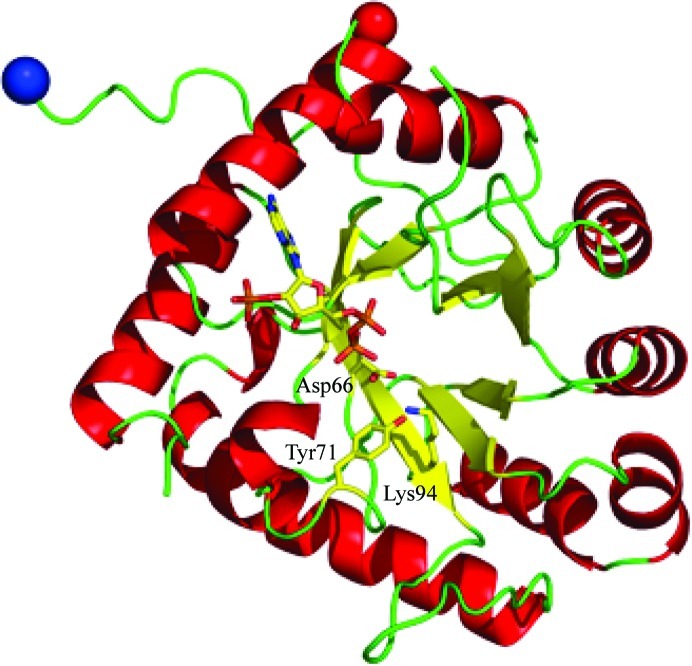
Schematic view of the structure of the putative aldo–keto reductase PA4992. Bound NADP^+^ and the putative catalytic triad are shown as stick models. The nicotinamide ribose moiety of NADP^+^ is not shown as it is disordered in the crystals.

PA4992 folds into a (β/α)_8_-barrel typical of aldo–keto reductases (Fig. 3 ▶ and Supplementary Fig. S1*c*). The closest structural homologue is AKR11C1 from *B. halodurans* (Marquardt *et al.*, 2005 ▶), with an r.m.s.d. of 1.8 Å for 220 aligned C^α^ atoms. Binding of NADP^+^ to PA4992 occurs in the open shallow crevice near the C-terminal helix α8, without significant conformational changes, as indicated by the r.m.s.d. of 0.3 Å between the structures of the apoenzyme and the holoenzyme. The adenosine segment of NADP^+^ is well defined in the electron-density map; however, the nicotinamide moiety is not and appears to be flexible. The position of the cofactor is similar to that observed in AKR11, although none of the residues forming hydrogen bonds or salt bridges with NADP^+^ atoms are retained in PA4992. Nevertheless, the enzyme contains the catalytic triad, in this case Asp66, Tyr71 and Lys94, which is conserved in the AKR family (Jez & Penning, 2001 ▶), suggesting a similar chemistry and mechanism.

### 3.7. Crystal structure of the conserved hypothetical protein PA4485

PA4485 is annotated as an essential gene in *P. aeruginosa* with no human homologue.

#### 3.7.1. Experimental

The gene coding for PA4485 was cloned into pEHISGFPTEV (Supplementary Table S2), expressed in *E. coli* and purified following the procedures outlined in §[Sec sec2]2. The construct entering our pipeline was truncated to residues 32–125. The crystals used for data collection were obtained using the sitting-drop vapour-diffusion method at 293 K by mixing 0.15 µl protein solution (8 mg ml^−1^) with 0.15 µl reservoir solution [25%(*w*/*v*) PEG 3350, 0.2 *M* sodium chloride, 0.1 *M* bis-tris pH 5.5]. The drops were equilibrated against 0.07 ml reservoir solution. Two data sets were collected: the first, for structure solution, was collected in-house and an additional higher resolution data set was collected on beamline I03 at Diamond Light Source and was used for refinement. For phasing, the crystals were soaked in 200 m*M* 5-amino-2,4,6-triiodoisophthalic acid (I_3_C) for approximately 5 min and were back-soaked in mother liquor containing 20%(*w*/*v*) glycerol prior to data collection. All data were processed and reduced with *HKL*-2000 (Otwinowski & Minor, 1997 ▶; Supplementary Table S6). Phases were determined using SAD (Sheldrick, 2008 ▶) and the structure was refined using the methods outlined in §[Sec sec2]2 (Table S6). The crystallographic data have been deposited in the PDB with accession code 4avr.

#### 3.7.2. Overall structure

**Figure 4:**
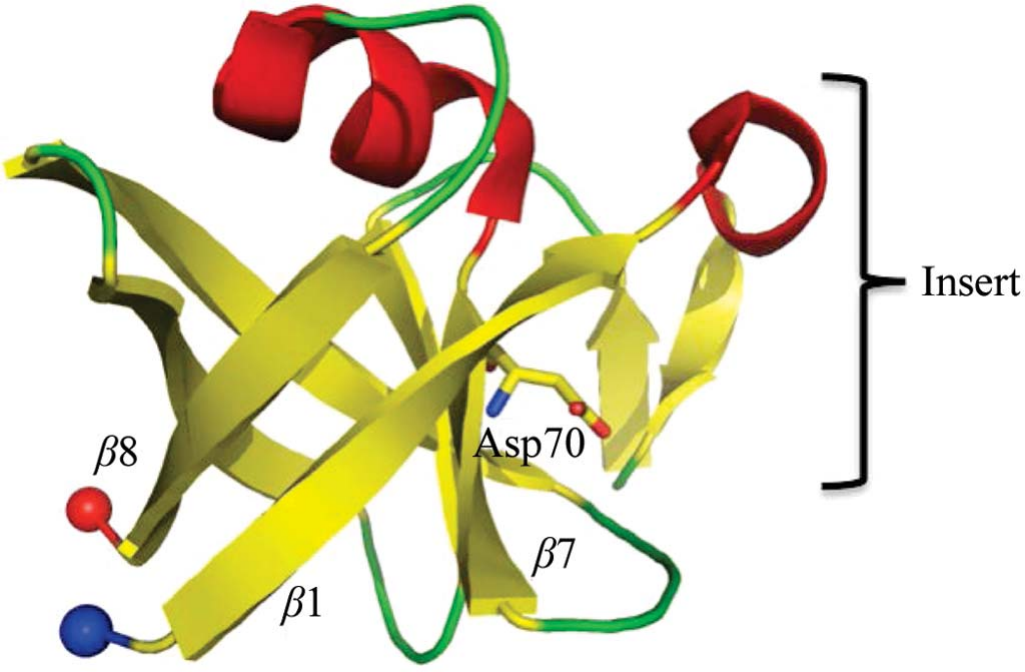
Schematic view of the structure of the uncharacterized protein PA4485. The conserved Asp70 is represented as a stick model.

PA4485 is a single-domain protein with overall dimensions of 25 × 23 Å. The core of the protein (residues 1–­9 and 26–94) adopts a six-stranded β-barrel fold (Fig. 4 ▶ and Supplementary Fig. 1*d*). The barrel is sealed off at one end by a seven-residue α-helix positioned between strands β7 and β8. In addition, there is a small extension protruding from the bulk of the protein (residues 10–25). After strand β1 of the barrel fold, this insert folds into a short 3_10_-helix that turns through almost a right angle such that the remaining two short β-strands pack against the core of the protein (Fig. 4 ▶).

The asymmetric unit of PA4485ΔN31 contains two molecules. Analysis with *PISA* suggests there is no stable dimer arrangement in the crystal. The *PISA* complex significance score (CSS) is 0.46, where a score of 1 suggests strong evidence for stable oligomer formation and a score of 0 represents little evidence of stable interfaces. The *PISA* analysis is consistent with the data from gel filtration, which indicate a monomer in solution.

The closest structural relatives of PA4485, with r.m.s.d. values in the range 1.9–2.0 Å, are Expb1, a β-expansin promoting extension and relaxation of grass cell walls (Yennawar *et al.*, 2006 ▶), MltA, a lytic transglycosylase that cleaves the β-1,4-glycosidic linkage between *N*-­acetylmuramic acid and *N*-acetylglucosamine of peptidoglycan (Powell *et al.*, 2006 ▶), and EGV, an endoglucanase responsible for hydrolysis of the β-1,4-linked glucose residues of cellulose (Hirvonen & Papageorgiou, 2003 ▶). All of these proteins belong to a family that shares the six-stranded double-Ψ β-barrel fold. Sequence alignments show that a catalytic aspartic acid, Asp70 in PA4485, is conserved across these proteins and also in PA4485, raising the possibility that this protein might have a similar function in polysaccharide hydrolysis. To investigate this hypothesis, native crystals were soaked with a variety of sugars including sucrose, arabinose, fructose and cellobiose, and X-ray diffraction data were subsequently collected and analysed. However, none of these experiments yielded electron-density maps that indicated the binding of a saccharide ligand.

### 3.8. Crystal structure of the hypothetical protein PA4098

PA4098 is an essential protein of *P. aeruginosa* and has been annotated as a probable short-chain dehydrogenase reductase (SDR).

#### 3.8.1. Experimental

The gene coding for PA4098 was cloned into pDEST14 (Supplementary Table S2), expressed in *E. coli* and purified following the procedures outlined in §[Sec sec2]2. The construct entering crystallization trials comprised residues 1–241. An additional glycine residue is present at the N-terminus of the protein owing to the cloning strategy. The hexagonal crystals used for data collection were obtained by the vapour-diffusion method at 293 K by mixing 1 µl protein solution (22 mg ml^−1^) with 1 µl reservoir solution [1.31 *M* sodium acetate, 0.14 *M* ammonium tartrate, 2%(*v*/*v*) butanediol, 0.1 *M* sodium acetate pH 4.5] and equilibrating the drops against 0.07 ml reservoir solution. Data were also collected from a complex of PA4098 with NAD^+^ obtained by soaking apo crystals grown from 1.56 *M* sodium acetate, 0.1 *M* ammonium tartate, 3.2%(*v*/*v*) butanediol, 0.1 *M* sodium acetate pH 5.0 in a cryobuffer consisting of 25%(*v*/*v*) PEG 400, 10 m*M* NAD^+^. The crystals were cryoprotected by soaking them in mother liquor supplemented with 15%(*v*/*v*) glycerol prior to data collection. X-ray data were collected on beamlines I03 and I02 at Diamond Light Source from crystals of the apoenzyme and the holoenzyme, respectively. X-ray data from the apoenzyme crystals were processed and scaled using *HKL*-2000 and data from the holoenzyme crystals were processed and scaled with *xia*2 (Supplementary Table S7).

Phases were obtained by MR using a monomer of 2-deoxy-d-gluconate 3-dehydrogenase from *Thermus thermophilus* (PDB entry 1x1e; RIKEN Structural Genomics/Proteomics Initiative, unpublished work) as a template and the structure was refined using *REFMAC*5 (Murshudov *et al.*, 2011 ▶). The asymmetric unit contained two molecules and all of the residues of the apo structure have been modelled satisfactorily despite relatively weak electron density for the loop between residues 182 and 188 (Supplementary Table S7). The crystallographic data have been deposited in the PDB with accession codes 4avy (apoenzyme) and 4b79 (holoenzyme).

#### 3.8.2. Overall structure

**Figure 5:**
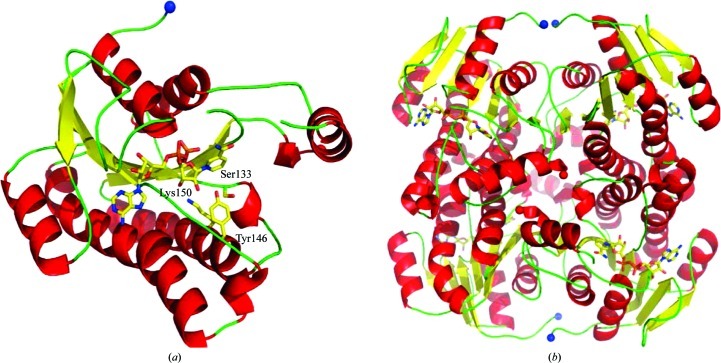
Schematic view of the structure of the putative short-chain dehydrogenase PA4098. (*a*) Structure of the enzyme subunit, with bound NAD^+^ and the putative catalytic triad Ser133, Tyr146 and Lys150 shown as stick models. (*b*) Tetrameric quaternary structure of PA4098.

PA4098 contains a Rossmann fold (Fig. 5 ▶ and Supplementary Fig. S1*e*), with the core of the subunit formed by a central parallel β-sheet of seven strands which is flanked by five α-­helices. The closest structural relative in the PDB is the hypo­thetical protein TT0321 from *T. thermophilus* HB8 (PDB entry 2d1y; 43% identity; RIKEN Structural Genomics/Proteomics Initiative, unpublished work), with an r.m.s.d. of 1.2 Å for 220 aligned C^α^ atoms. The major structural difference between PA4098 and this and other closely related members of this enzyme family is the lack of an α-helix between the β2 and β3 strands in PA4098. The overall structures of the polypeptide chains in the asymmetric units are almost identical, with r.m.s.d. values of 0.2 Å (apo *versus* apo) and 0.3 Å (apo *versus* holo). PA4098 forms a tetramer in the crystal similar to the subunit arrangement in other family members.

The binding of the cofactor NAD^+^ at the end of the central β-sheet is similar to that observed in other SDR enzymes (Oppermann *et al.*, 2003 ▶). Binding of NAD^+^ results in a different conformation of the loop comprising residues 182–188 (involved in NAD^+^ binding), which becomes partially disordered in the holo structure. A sequence alignment of PA4098 with SDR family members shows that all of the sequence motifs common to SDR enzymes and required for NAD binding and activity are conserved in PA4098 and the proposed catalytic triad of PA4098, Ser133, Tyr146 and Lys150, adopts a similar conformation and displays interactions with those observed in other SDR enzymes. The presence of an acidic residue in the pocket would preclude the binding of NADP (Persson *et al.*, 2003 ▶) owing to clashes with the phosphate group, while its absence and a basic residue close by indicates NADP binding. There are no such basic or acidic residues in the sequence of PA4098; instead, the 2-OH group forms a hydrogen bond to the backbone of Leu42. This region of the protein structure would preclude binding of NADP (clash with phosphate) without significant rearrangement of the structure.

### 3.9. Crystal structure of PA3770

PA3770 is annotated as inosine-5′-monophosphate dehydrogenase (IMPDH) catalyzing the conversion of inosine 5′-monophosphate to xanthosine 5′-monophosphate in the guanine-nucleotide biosynthesis pathway.

#### 3.9.1. Experimental

The gene coding for PA3770 was cloned into pDEST14 (Supplementary Table S2), expressed in *E. coli* and purified following the procedures outlined in §[Sec sec2]2. The construct entering crystallization trials comprised residues 1–489 of the PA3770 coding sequence with an additional glycine residue at the N-terminus arising from the cloning protocol. The crystal used for data collection was obtained by sitting-drop vapour diffusion at 293 K by mixing 0.15 µl protein solution (13 mg ml^−1^) with 0.15 µl reservoir solution [0.1 *M* MES pH 6.5 containing 11%(*w*/*v*) PEG 4000]. Droplets were equilibrated against 0.07 ml reservoir solution. The crystals were cryoprotected by doping the mother liquor with 25%(*v*/*v*) glycerol prior to data collection. X-ray data were collected on beamline ID29 at ESRF and were processed using *xia*2 (Winter, 2010 ▶; Supplementary Table S8).

Phases were obtained by MR using the structure of a subunit of inosine-5′-monophosphate dehydrogenase from *Thermotoga maritima* (PDB entry 1vrd; Joint Center for Structural Genomics, unpublished work) as a template and the structure of PA3770 was refined using the protocol described in §[Sec sec2]2 (Supplementary Table S8). The crystallographic data have been deposited in the PDB with accession code 4avf.

#### 3.9.2. Overall structure

**Figure 6:**
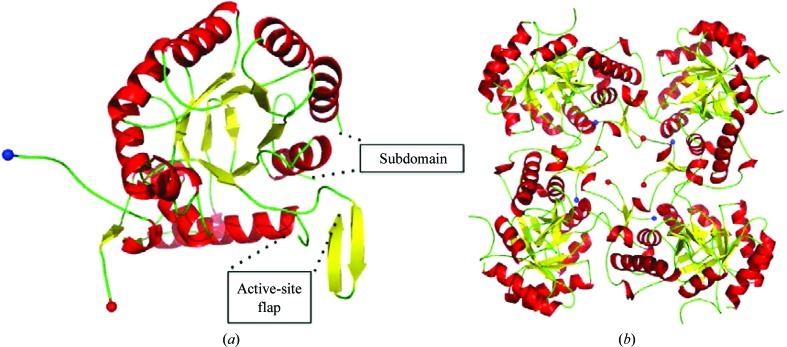
Schematic view of the structure of the subunit (*a*) and tetramer (*b*) of the putative inosine-5′-monophosphate dehydrogenase PA3770. The location of the missing subdomain and active-site flap are highlighted in the structure of the subunit.

The three-dimensional structure of IMPDH has been well characterized and has recently been reviewed (Hedstrom, 2009 ▶). The catalytic domain folds into an eight-stranded β/α-barrel (Fig. 6 ▶ and Supplementary Fig. S1*f*). The closest structural homologue to PA3770 is IMPDH from the Gram-negative bacterium *Borrelia burgdorferi* (PDB entry 1eep; McMillan *et al.*, 2000 ▶). The two proteins align over 313 residues with an r.m.s.d. of 0.8 Å. Like many of the IMPDH structures deposited in the PDB, no electron density is visible in our structure for the subdomain residues 91–204. In addition, electron density for residues 385–420 corresponding to the so-called ‘active-site flap’ is also missing. Nevertheless, the overall architecture of the proposed active site in PA3770 is the same as that found in the *B. burgdorferi* enzyme.

The protein forms a tetramer in the asymmetric unit with a total buried surface area of 11 200 Å^2^, equivalent to approximately 23% of the total surface area. Data from gel filtration indicated a molecular mass of around 200 kDa, which is consistent with tetramer formation.

### 3.10. Crystal structure of the hypothetical protein PA1645

PA1645 is annotated as a hypothetical protein that has no human homologues.

#### 3.10.1. Experimental

The gene coding for PA1645 was cloned into pEHISGFPTEV (Supplementary Table S2), expressed in *E. coli* and purified following the procedures outlined in §[Sec sec2]2. The target entering our pipeline was truncated to residues 20–135. The crystal used for data collection was obtained by sitting-drop vapour diffusion at 293 K by mixing 0.15 µl protein solution (6 mg ml^−1^) with 0.15 µl reservoir solution (0.64 *M* lithium sulfate, 0.18 *M* sodium acetate, 0.1 *M* sodium citrate pH 4.5). The drops were equilibrated against 0.07 ml reservoir solution. The crystals were cryoprotected by doping the mother liquor with 25%(*v*/*v*) glycerol prior to data collection. Extremely highly redundant X-ray data were collected at a wavelength of 1.6 Å on beamline I03 at Diamond Light Source and were processed with *xia*2 (Winter, 2010 ▶; Supplementary Table S9).

Phases were determined by S-SAD using the *SHELXC*/*D*/*E* suite of programs (Sheldrick, 2008 ▶) and the structure was refined using *REFMAC*5 (Murshudov *et al.*, 2011 ▶; Supplementary Table S9). All of the residues are ordered in each of the three monomers in the asymmetric unit. The crystallographic data have been deposited in the PDB with accession code 2xu8.

#### 3.10.2. Overall structure

**Figure 7:**
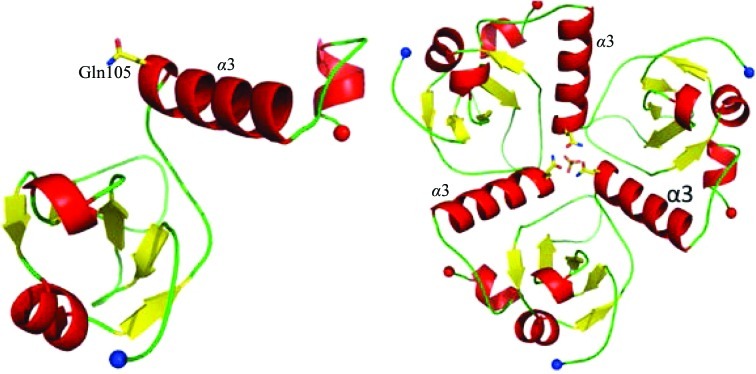
Schematic view of the structure of the monomer and the putative trimer of the uncharacterized protein PA1645. Helices α3 that form a ‘funnel’-like structure are labelled alongside Gln105, which blocks the entrance of this funnel. The sulfate ion at the base of the funnel is also depicted.

Each monomer consists of a five-stranded antiparallel β-sheet sandwiched between two short α-helices (Fig. 7 ▶ and Supplementary Fig. S1*g*). One large loop protrudes between strands 3 and 4, and in the trimer these loops form an apex to the oligomeric structure. On the opposite side of the trimer, α3 from each monomer packs in a manner akin to an angled propeller, creating a funnel leading to a positively charged surface at the base. However, the funnel is partially blocked by residue Gln105. PA1645 also binds 11 SO_4_^2−^ ions, a component of the crystallization conditions. Interestingly, one of the SO_4_^2−^ ions is located just below the triad of Gln105 residues and is anchored by a network of interactions with water molecules. There are no direct contacts between the sulfate ion and the protein.

An analysis of the oligomerization state of the protein with *PISA* returned a CSS of only 0.194. Such a low score suggests that the proposed arrangement may not be stable. The total buried surface area in the PA1645ΔN19 trimer is 8230 Å^2^, which represents 46% of the total surface area available. This compares with a total buried surface area of 6320 Å^2^, which is 34% of the total surface area available, for *Plasmodium falciparum* dUTPase (PDB entry 2y8c), a confirmed protein trimer of similar mass to PA1645 (Baragaña *et al.*, 2011 ▶). However, PA1645ΔN19 elutes from a gel-filtration column as two peaks, one of which is analogous in size to the proposed trimer, with a molecular mass close to 40 kDa. These data therefore suggest that PA1645 most likely exists in solution in an equilibrium between monomeric and trimeric states.

### 3.11. Crystal structure of the putative oxidoreductase PA1648

PA1648 has been annotated as a probable oxidoreductase.

#### 3.11.1. Experimental

The gene coding for PA1648 was cloned into pDEST14 (Supplementary Table S2), expressed in *E. coli* and purified following the procedures outlined in §[Sec sec2]2. The construct entering crystallization trials comprised residues 1–334 encoded by the PA1648 gene and one additional glycine residue at the N-terminus of the protein as a result of the cloning protocol. The crystal used for data collection was obtained by mixing 1 µl reservoir solution [0.9 *M* sodium citrate, 0.1 *M* MES pH 6.5, 0.1 *M* magnesium sulfate, 10%(*v*/*v*) glycerol] with 1 µl protein solution and equilibrating against 0.1 ml well solution. The crystal was cryoprotected by soaking in mother liquor containing 20%(*v*/*v*) glycerol prior to data collection. In addition, an NADP^+^-bound complex was formed by soaking a native crystal with 25 m*M* NADP^+^ overnight prior to cryoprotection and data collection. X-ray data were collected on beamlines I03 and I02 at Diamond Light Source from crystals of the apoenzyme and the holoenzyme, respectively. X-ray data were processed with *XDS* and scaled using *XSCALE* (Kabsch, 2010 ▶; Supplementary Table S10).

Phases were determined by MR using a model generated from double-bond reductase from *Arabidopsis thaliana* (Youn *et al.*, 2006 ▶; PDB entry 2j3h). The apo structure was used as a model to solve the ligand-bound complex. The protein models were refined using *REFMAC*5 (Murshudov *et al.*, 2011 ▶) and protocols described in §[Sec sec2]2. Details of the refinement statistics are given in Supplementary Table S10. The crystallographic data have been deposited in the PDB with accession codes 4b7c (apoenzyme) and 4b7x (holoenzyme).

#### 3.11.2. Overall structure

**Figure 8:**
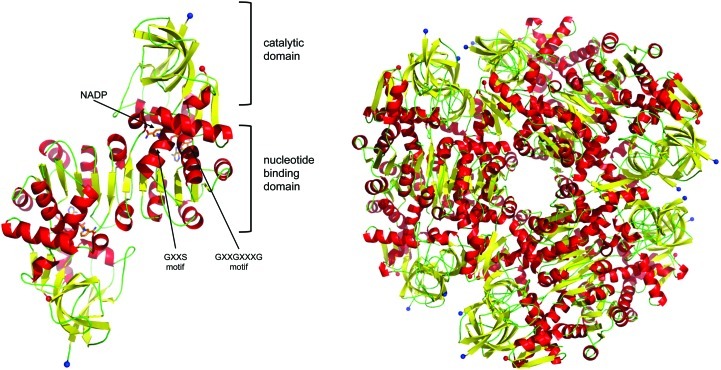
Schematic view of the dimer (*a*) and dodecamer (*b*) of the probable oxidoreductase PA1648. The catalytic and nucleotide-binding domains are labelled alongside the two consensus motifs and the NADP^+^-binding site.

The PA1648 monomer is comprised of a catalytic domain and a nucleotide-binding domain (Fig. 8 ▶ and Supplementary Fig. S1*h*). The catalytic domain is formed by residues 1–129 and 299–334, which includes α-helices 1, 2 and 11 and β-strands 1–8, 15 and 16. The strands form five-stranded and three-stranded antiparallel twisted sheets and a two-stranded parallel β-sheet. The nucleotide-binding domain comprises residues 130–298 (α-helices 3–­10 and β-strands 9–14) and adopts the characteristic Rossmann fold. The cofactor-binding site is located in a cleft between the catalytic and nucleotide-binding domains. A *DALI* search of the PDB with the PA1648 dimer revealed structural similarity to numerous members of the medium-chain reductase superfamily. The conservation of sequence is most pronounced in the nucleotide-binding domain and includes the G*XX*S (residues 246–249) and glycine-rich G*XX*G*XXX*G (residues 158–165) motifs, which interact with the adenine and nicotinamide moieties of the NADP^+^, as observed in the homologue 2j3h (Youn *et al.*, 2006 ▶). This particular homologue shares a sequence identity of 41% with PA1648 and aligns 326 C^α^ atoms with an r.m.s.d. of 1.4 Å. The catalytic domain showed relatively little sequence identity (5%) to the structural homologues and lacks the polyproline helix observed in some other members of the family.

There are 12 molecules in the asymmetric unit, which form six homodimers assembled in a trimeric arrangement (Fig. 8 ▶). Gel-filtration analyses show the protein to be dimeric in solution, suggesting that the dodecamer arrangement is a crystallographic artifact.

## 4. Concluding remarks

Our long-term objective is to elucidate the potential and to provide a comprehensive assessment of selected *P. aeruginosa* proteins as targets for therapeutic intervention. In this endeavour, we targeted a number of proteins for crystallographic study. We generated valuable reagents such as expression plasmids for efficient production of soluble recombinant proteins and established protocols for purification, biochemical and biophysical assays, compound screening and crystallization. We have obtained new structural data on potential drug targets and enriched the PDB collection of structures from this pathogen. The structural information that we have generated has allowed an assessment of the druggability of these targets based on a novel algorithm (Krasowski *et al.*, 2011 ▶) and will be published elsewhere. Furthermore, our work provides templates for structure-based approaches by computational methods and/or fragment screening to further support inhibitor development. New chemical entities that bind and inhibit selected targets have already been identified and will provide starting points for hit-to-lead compound development. Our PAO1 genome assessment, results and experiences are available online at http://aeropath.lifesci.dundee.ac.uk/.

## Supplementary Material

Supplementary material file. DOI: 10.1107/S1744309112044739/wd5195sup1.pdf

PDB reference: 4es6

PDB reference: 4etr

PDB reference: 4exb

PDB reference: 4exa

PDB reference: 4avr

PDB reference: 4avy

PDB reference: 4b79

PDB reference: 4avf

PDB reference: 2xub

PDB reference: 4b7c

PDB reference: 4b7x

## Figures and Tables

**Table 1:** Summary of gene-to-structure statistics within the AEROPATH project

Targets	Cloned	Expressed	Soluble	Insoluble	Purified	Crystals	Structures
Total	102	99	84	18	79	47	39†

† Includes two NMR structures.

**Table 2:** Phasing experiments for the crystal structures

	MR	S-SAD	Se-SAD	HA-SAD	Se-MAD
Total	24	6	2	4	1

## Footnotes

1 Supplementary material has been deposited in the IUCr electronic archive (Reference: WD5195).
