# Supplementary material for: The AEROPATH project targeting Pseudomonas aeruginosa: crystallographic studies for assessment of potential targets in early-stage drug discovery
Source: Acta Crystallogr Sect F Struct Biol Cryst Commun. 2012 Dec 25;69(Pt 1):25–34. doi: 10.1107/S1744309112044739 (PMC3539698; doi:10.1107/S1744309112044739)

Supplementary material

Supplementary table 1: AEROPATH targets from *Pseudomonas aeruginosa* and closely related Gram-negative bacteria.

	**Annotation**	**# AA**	**Soluble protein**[a]	**Crystals**	**PDB entry**
PA0008	glycyl-tRNA synthetase beta chain	684	Yes	No	
PA0009	glycyl-tRNA synthetase alpha chain	315	Yes	No	
PA0008/0009 complex	glycyl-tRNA synthetase alpha and beta chain	684 + 315	Yes	Yes	
PA0080	Lipoprotein associated with type 6 secretion system; Serratia marcescens orthologue	149	Yes	Yes	4AIR
PA0223	putative dihydropicolinate synthase	293	Yes	No	
PA0254	putative aromatic decarboxylase, UbiD homologue	496	Yes	Yes	
PA0342	thymidylate synthase	264	Yes	No	
PA0408	twitching motility protein PilG	135	No	No	
PA0503	probable biotin synthesis protein BioC	274	No	No	
PA0517Δ19	probable c-type cytochrome precursor	100	Yes	No	

MBP-PA0655[b]	probable ubiquitin biosynthesis enzyme (CoQ7 homologue)	215	Yes	No	
PA0656	probable diadenosine tetraphosphate hydrolase (HIT domain protein)	112	Yes	No	
PA0856	hypothetical protein	182	Yes	Yes	2X3O
PA1000	quinolone signal response protein	301	Yes	Yes	2VW8
PA1010	dihydropicolinate synthase	292	Yes	Yes	3QZE
PA1075	hypothetical protein	105	Yes	No	2LV5 solved by NMR
PA1165	siderophore biosynthesis protein	242	No	No	
PA1456	response regulator CheY	124	No	No	
PA1610	hydroxydecanoyl thiolester dehydratase	171	Yes	Yes	4FQ9
GFP-PA1645ΔN19[b]	hypothetical protein	116	Yes	Yes	2XU8
PA1648	probable oxido-reductase	334	Yes	Yes	4B7C, 4B7X
PA1681	chorismate synthase	363	Yes	No	
PA1697	putative ATP synthase component	440	No	No	
PA1792	putative UDP-2,3-diacylglucosamine hydrolase	240	Yes	No	

PA1793	putative PPIase	165	Yes	No	
PA1796	5,10-methylene-tetrahydrofolate dehydrogenase / cyclohydrolase (FolD)	284	Yes	Yes	4A5O
PA1806[c]	NADH-dependent enoyl-ACP reductase (FabI)	265	Yes	No	
PA1837	hypothetical protein	165	Yes	No	
PA1844	peptidoglycan amidase	154	Yes	Yes	
PA2048	hypothetical protein	135	Yes	No	
PA2056	transcriptional regulator	300	No	No	
PA2081	kynurenine formamidase (KynB)	209	Yes	Yes	
PA2086	putative epoxide hydrolase	300	Yes	Yes	4B9A, 4B9E
PA2143	hypothetical protein	95	Yes	No	
PA2149	hypothetical protein	80	Yes	No	
PA2169	hypothetical protein	150	Yes	Yes	4ETR
PA2273	SoxR	156	Yes	No	
PA2497	Immunity protein; S. marcescens orthologue	119	Yes	Yes	4AX2

PA2537	probable acyltransferase	209	No	No	
PA2655	hypothetical protein	141	No	No	
PA2831	metallocarboxypeptidase	384	Yes	Yes	
PA2950<br><br>Bc homologue[d]	putative enoyl-acyl carrier protein (ACP) reductase (FabV)	398<br><br>400	Yes<br><br>Yes	No<br><br>Yes	
PA2964	4-amino-4-deoxychorismate lyase	271	Yes	Yes	2Y4R
PA2965	ketoacyl carrier protein synthase II (FabF)	414	Yes	Yes	4B7V
PA2967	3-oxo-acyl-ACP reductase, FabG	247	Yes	Yes	4AFN, 4AG3
PA2977	UDP-N-acetylpyruvoyl glucosamine reductase	339	Yes		
PA2981	tetraacyldisaccharide 4’-kinase	332	No	No	
PA2987	probable ATP-binding component of ABC transporter	227	No	No	
PA3155	UDP-2-acetamido-2-dideoxy-D-ribo-hex-3-uluronic acid transaminase, wbpE	359	Yes	Yes	
PA3171	putative 3-demethylubiquinone-9,3-methyltransferase (UbiG)	232	Yes	Yes	
PA3333	3-oxoacyl-ACP synthase (FabH)	330	Yes	Yes	2X3E

PA3528	ribonuclease T	224	Yes	Yes	2F96[e]
PA3643	lipid-A disaccharide synthase	378	Yes	No	
PA3648	outer membrane protein Opr86	797	No	No	
PA3650	deoxy-xylulose-5-phosphate reductoisomerase	398	Yes	Yes	
PA3666	tetrahydrodipicolinate N-succinyl -transferase (DapD)	344	Yes	Yes	3R5A, 3R5B, 3R5C, 3R5D
PA3770	inosine-5-monophosphate dehydrogenase	489	Yes	Yes	4AVF
PA3793	hypothetical protein	110	Yes	No	Solved by NMR
PA3803	4-hydroxy-3-methylbut-2-en-1-yl diphosphate synthase	371	Yes	No	
PA4019	putative aromatic decarboxylase	209	Yes	Yes	3ZQU
PA4043	putative geranyltransferase	295	Yes	Yes	
PA4051	thiamine monophosphate kinase	322	No	No	
PA4056	riboflavin-specific deaminase/ reductase	361	Yes	Yes	
PA4068	probable epimerase	309	Yes	Yes	

PA4090	hypothetical protein	96	Yes	No	2JPl[f]
PA4098	probable short-chain dehydrogenase, NAD^+ complex	241	Yes	Yes	4AVY 4B79
PA4115	Immuniy protein type 6 secretion S. marcescens orthologue	119	Yes	Yes	4B6I
PA4188	probable dihydropicolinate synthase (DapA)	303	Yes	No	
PA4190	putative monooxygenase	398	Yes	Yes	2X3N
PA4389	arginine decarboxylase	252	Yes	Yes	
PA4406	UDP-3-O-acyl-N-acetylglucosamine deacetylase	301	No	No	
PA4416	UDP-N-acetylmuramoyl-tripeptide--D-alanyl-D-alanine ligase	458	Yes	No	
PA4417	UDP-N-acetylmuramoylalanyl-D-glutamate--2,6-diaminopimelate ligase	487	Yes	No	
PA4450	UDP-N-acetylglucosamine 1-carboxyvinyltransferase	419	Yes	No	
PA4460	hypothetical protein	175	Yes	No	
GFP-PA4485ΔN31	hypothetical protein	94	Yes	Yes	4AVR
PA4511	hypothetical protein	251	Yes	Yes	2X5E
PA4557	4-hydroxy-3-methylbut-2-enyl diphosphate reductase (LytB)	314	Yes	No	

PA4631	hypothetical protein	341	Yes	Yes	2X4G
PA4636	probable glycerol-3-phosphate O-acyltransferase	386	Yes	No	
PA4655	ferrochelatase (HemH)	340	Yes	No	
PA4662[b]	glutamate racemase	265	Yes	Yes	
PA4679	hypothetical protein	233	No		
PA4715	putative aminotransferase	411	Yes	Yes	2X5D
PA4750	dihydropteroate synthase B. cenocepacia orthologue	263	Yes	Yes	2Y5S
PA4848	biotin carboxylase	446	Yes	No	
PA4918	nicotinamidase, A. baumanni orthologue	219	Yes	Yes	2WTA
PA4931[c]	replicative DNA helicase	464	Yes	No	
MBP-PA4988[b]	probable 3-deoxy-D-manno-octulosonic-acid transferase	425	Yes	Yes	
PA4991	FAD dependent oxidoreductase	391	Yes	Yes	
PA4992	putative aldo-keto reductase	270	Yes	Yes	4EXA, 4EXB
PA4998	hypothetical protein	216	Yes	No	

PA5006	hypothetical protein	492	No	No	
PA5038	3-dehydroquinate synthase	368	Yes	No	
PA5063	ubiquinone biosynthesis methyl transferase UbiE	256	Yes	No	
PA5065	ubiquinone biosynthetic protein UbiB	533	No	No	
PA5119	glutamine synthetase	469	Yes	Yes	
PA5174	probable keto-acyl synthase	634	Yes	No	
PA5237	putative aromatic decarboxylase UbiD	488	Yes	Yes	
PA5259	uroporphyrinogen-III synthetase (HemD)	254	Yes	Yes	4ES6
PA5321	deoxyuridine-5'-triphosphate nucleotidohydrolase	151	No	No	
PA5407	hypothetical protein	96	Yes	No	

a. soluble in small scale expression screen; "No" means either no expression or insoluble material. b: fusion constructs with maltose binding protein (MBP) or green fluorescent protein (GFP); c:multiple construct approach was required, d: *Burkholderia cenocepacia*; e. structure determined by Zuo, Y., Zheng, H., Wang, Y., Chruszcz, M., Cymborowski, M., Skarina, T., Savchenko, A., Malhotra, A. & Minor, W. (2007) *Structure* **15**, 417-428; f. structure determined by Ai, X.,Semesi, A.,Yee, A., Arrowsmith, C.H., Choy, W. & Li, S. (to be published).

Table S2: Amplification primers and protein constructs used for structure determination.

Target	Upstream amplification primer (restriction sites undelined)	Downstream amplification primer(restriction sites undelined)	Expression tag (*cleavage position)	Construct used in crystallization
**PA1645**	AGGGCGCCATGGACTGGAGCGGCC CGATCGAGCAG	GAATTCGGATCCTCAGTCCGGCG CCCGCAGGCACTC	N'-MSYYHHHHHHDYD(e**GFP**)DYDIPTTENLYFQ* GAM	His6-GFP tag removed, residual: N'-GAM
**PA1648**	CCGAAAACCTGTATTTTCAGGGCAT GACCTCCCAGATCAATCGGCAATAC	GGGGACCACTTTGTACAAGAAAG CTGGGTCCTAGACCTTCAACACC AACTTGCCG	N'-MSYYHHHHHHDYDIPTTENLYFQ*GM	His6-tag removed, residual: N'-G
**PA2169**	GATCACATATGAACCAGACCAACCT CGAC	AGTCAAGCTTAGGCGCGGGCGTC GCGCA	N'-MGSSHHHHHHSSGLVPR*GSH	His6-tag removed, residual: N'-GSH-
**PA3770**	CCGAAAACCTGTATTTTCAGGGCAT GCTGCGAATCAGTCAAGAAGCCC	GGGGACCACTTTGTACAAGAAAG CTGGGTCCTAACCAACCCGGTAG TTGGGGGC	N'-MSYYHHHHHHDYDIPTTENLYFQ*GM	His6-tag removed, residual: N'-G
**PA4098**	CCGAAAACCTGTATTTTCAGGGCAT GGTTTTCCAACACGATATCTACGCC	GGGGACCACTTTGTACAAGAAAG CTGGGTCCTAGGCGCAGAGATAG CCGCCGTC	N'-MSYYHHHHHHDYDIPTTENLYFQ*GM	His6-tag removed, residual: N'-G
**PA4485**	AGGGCGCCATGGATACCGGCGAAG CCTCCTACTATG	GAATTCGGATCCTCAATCCAGCG ACTCTATCCGAACCG	N'-MSYYHHHHHHDYD(e**GFP**)DYDIPTTENLYFQ* GAM	His6-GFP tag removed, residual: N'-GAM
**PA4992**	TACTTCCAATCCATGATCAGGGATA CCCTCCACG	TATCCACCTTTACTGTCAGGCCTT TTTCAGGGCTTGC	N'-MHHHHHHSSGVDLGTENLYFQ*S	His-tagged construct
**PA5259**	GATCACATATGAGCGGCTGGCGGCT CC	AGTCAAGCTTAGGCGGCGCTCGT CAGGG	N'-MGSSHHHHHHSSGLVPR*GSH	His6-tag removed, residual: N'-GSH-

Table S3: Data collection and refinement statistics for PA5259.

	**PA5259 - native**	**PA5259 Se-SAD**
**Data collection**		
Beam line	ID23:1, ESRF	ID14-4, ESRF
Wavelength (Å)	0.9794	0.9785
Space group	$P4_32_12$	$P4_32_12$
Cell dimensions		
a,, c (Å)	132.4, 42.5	132.3, 42.5
Resolution (Å)*	66.0-2.22 (2.34-2.22)	66.2 – 2.28 (2.40-2.28)
Total no. of reflections	119166	246762
Unique reflections	18993	18032
Redundancy	6.3 (4.3)	13.7 (13.9)
Completeness (%)	99.0 (95.5)	99.9 (100)
Mean $I/\sigma(I)$	12.0 (3.1)	15.5 (4.5)
R_{merge}	0.084 (0.539)	0.098 (0.538)
Solvent (%)	65	65
**Refinement**		
R_{work} / R_{free} (%)	21.2/ 25.2 (38.1/45.2)	
r.m.s.d. Bonds (Å)/Angles (°)	0.010 / 1.17	
No. atoms		
Protein	1913	
Solvent	100	
B factors (Å^2)		
Protein	39.2	
Water	43.5	
Ramachandran plot		
Allowed/disallowed (%)	98.0/0	
Molprobity score / percentile	1.76 (95)	
PDB code	4ES6	

*Values in parenthesis refer to the highest resolution shell

Table S4: Data collection and refinement statistics for PA2169.

	**PA2169**
**Data collection**	
Beam line	ID23:1, ESRF
Wavelength (Å)	0.9762
Space group	$P2_1$
Cell dimensions	
a, b, c (Å)	40.6, 71.2, 48.8
α, β, γ (°)	90.0, 111.6, 90.0
Resolution (Å)*	45.4-2.25 (2.37-2.25)
Total no. of reflections	41646
Unique reflections	11347
Redundancy	3.5 (3.5)
Completeness (%)	96.8 (96.2)
Mean $I/\sigma(I)$	8.5 (2.6)
R_{merge}	0.092 (0.48)
Solvent (%)	50
**Refinement**	
R_{work} / R_{free}	22.1/ 28.5 (37.5/41.0)
r.m.s.d. Bonds(Å)/Angles(°)	0.015 / 1.507
No. atoms	
Protein	1916
Water	21
B factors (Å^2)	
Protein	40.2
Water	39.2
Ramachandran	
Allowed/disallowed (%)	100/0
Molprobity score / centile	1.92 (90)
PDB code	4ETR

*Values in parenthesis refer to the highest resolution shell

Table S5: Data collection and refinement statistics for PA4992.

	**Apo-PA4992**	**PA4992–NADP+ complex**
**Data collection**		
Beam line	I9-11, MAX IV Laboratory	ID23:1, ESRF
Wavelength (Å)	0.999	0.8726
Space group	$P2_12_12_1$	$P2_12_12_1$
Cell dimensions		
a, b, c (Å)	94.3, 119.0, 150.8	94.2, 120.3, 151.1
Resolution (Å)*	42.1-2.75 (2.90-2.75)	94.6-2.80 (2.95-2.80)
Total no. of reflections	160685	170383
Unique reflections	44634	41558
Redundancy	3.6 (3.5)	4.1 (4.1)
Completeness (%)	99.6 (97.7)	97.2 (98.2)
Mean $I/\sigma(I)$	9.5 (2.0)	8.9 (2.7)
R_{merge}	0.103 (0.637)	0.129 (0.493)
Mean $I/\sigma(I)$	9.5 (2.0)	8.9 (2.7)
Solvent (%)	50	50
**Refinement**		
R_{work} / R_{free}	24.4/27.4 (36.6/37.8)	22.7/27.2 (33.5/35.6)
r.m.s.d. Bonds(Å)/Angles(°)	0.016 / 1.44	0.013 / 1.35
No. atoms		
Protein	11572	11624
Cofactor	-	186
Solvent	110	145
B factors (Å^2)		
Protein	37.1	30.5
Cofactor	-	42.0
Solvent	29.0	25.0
Ramachandran		
Allowed/disallowed (%)	95.2/0.1	95.4/0.34
Molprobity score / percentile	1.73 (98)	1.73 (98)
PDB code	4EXB	4EXA

*Values in parenthesis for all Tables refer to the highest resolution shell

Table S6: Data collection and refinement statistics for PA4485ΔN31.

	PA4485ΔN31 - native	PA4485ΔN31 – I_3C
**Data collection**		
Beam line	I03, Diamond	In house
Wavelength (Å)	0.97	1.54
Space group	*P*1	*P*1
Cell dimensions		
a, b, c (Å)	34.0, 39.0, 41.9	34.5, 38.2, 42.0
α, β, γ (°)	102.8, 103.5, 115.6	66.4, 79.7, 65.2
Resolution (Å)*	50 – 1.08 (1.1-1.08)	50 – 1.95 (1.98-1.95)
Total no. of reflections	282056	202620
Unique reflections	69440	12296
Redundancy	4. (3.6)	3.8 (2.4)
Completeness (%)	92.1 (88.8)	94.4 (82.9)
Mean $I/\sigma(I)$	38.7 (9.1)	41.4 (18.2)
R_{merge}	0.063 (0.168)	0.085 (0.11)
Solvent (%)	45	45
**Refinement**		
R_{work} / R_{free} (%)	14.8 / 17.7	
r.m.s.d. Bonds(Å)/Angles(°)	0.009 / 1.53	
No. atoms		
Protein	1462	
Solvent	204	
B factors (Å^2)		
Protein	15.4	
Water	29.8	
Ramachandran plot		
Allowed/disallowed (%)	98.4 / 0	
Molprobity score / percentile	0.93 / 99	
PDB code	4AVR	

*Values in parenthesis refer to the highest resolution shell

Table S7: Data collection and refinement statistics for PA4098.

	PA4098 - apo	PA4098 – NAD^+
**Data collection**		
Beam line	I03, Diamond	I02, Diamond
Wavelength (Å)	0.97	0.97
Space group	$P6_422$	$P6_422$
Cell dimensions		
a, b, c (Å)	105.7, 105.7, 204.9	103.9, 103.9, 205.5
α, β, γ (°)	90.0, 90.0, 120.0	90.0, 90.0, 120.0
Resolution (Å)*	36.78-2.00 (2.03-2.00)	67.70-1.98 (2.03-1.98)
Total no. of reflections	544209	600708
Unique reflections	44003	46652
Redundancy	12.3 (12.4)	12.9 (13.2)
Mean $I/\sigma(I)$	37.9 (5.4)	27.7 (4.1)
R_{merge}	0.083 (0.545)	0.066 (0.671)
Solvent (%)	60	60
**Refinement**		
R_{work} / R_{free} (%)	17.2 / 17.7	19.3 / 21.3
r.m.s.d. Bonds(Å)/Angles (°)	0.008 / 1.07	0.009 / 1.39
No. atoms		
Protein	3609	3533
Solvent	241	177
NAD^+		88
B factors ($Å^2$)		
Protein	26.8	34.1
Water	47.6	35.9
NAD^+		34.7
Ramachandran plot		
Allowed/disallowed (%)	97.0/0	97.0 / 0
Molprobity score / percentile	1.34 / 99	1.46/ 97
PDB code	4AVY	4B79

*Values in parenthesis refer to the highest resolution shell

Table S8: Data collection and refinement statistics for PA3770.

	PA3770
**Data collection**	
Beam line	ID29, ESRF
Wavelength (Å)	0.99
Space group	$P4_12_12$
Cell dimensions	
a, b, c (Å)	116.6, 259.2
Resolution (Å)*	106-2.23 (2.29-2.23)
Total no. of reflections	797080
Unique reflections	87809
Redundancy	9.1 (9.4)
Completeness (%)	100 (100)
Mean $I/\sigma(I)$	18.5 (3.2)
R_{merge}	0.075 (0.75)
Solvent (%)	40
**Refinement**	
R_{work} / R_{free}	19.2/21.9
r.m.s.d. Bonds(Å)/Angles(°)	0.012 / 1.243
No. atoms	
Protein	9146
Water	343
B factors (Å^2)	
Protein	26.2
Water	30.3
Ramachandran	
Allowed/disallowed (%)	97.6/0.3
Molprobity score / centile	1.57 (98)
PDB code	4AVF

*Values in parenthesis refer to the highest resolution shell

Table S9: Data collection and refinement statistics for PA1645ΔN19.

	**PA1645ΔN19**
**Data collection**	
Beam line	IO3, Diamond
Wavelength (Å)	1.6
Space group	$I4_122$
Cell dimensions	
a, c (Å)	125.9, 125.8
Resolution (Å)*	89 – 1.98 (2.03-1.98)
Total no. of reflections	2591494
Unique reflections	35545
Redundancy	72.9 (7.4)
Completeness (%)	99.9 (99.9)
Mean $I/\sigma(I)$	56.5 (9.4)
R_{merge}	0.086 (0.75)
Mean $I/\sigma(I)$	56.5 (9.4)
Solvent (%)	60
**Refinement**	
R_{work} / R_{free}	17.6/19.2
r.m.s.d. Bonds(Å)/Angles(°)	0.009 / 1.21
No. atoms	2851
Protein	2851
Water	246
Sulphate	11
B factors (Å^2)	
Protein	42.9
Water	46.9
Sulphate	59.3
Ramachandran	
Allowed/disallowed (%)	98.2/0.9
Molprobity score / centile	1.15 (100)
PDB code	2XU8

*Values in parenthesis refer to the highest resolution shell

Table S10: Data collection and refinement statistics for PA1648.

	**PA1648 - apo**	**PA1648 – NADP$^+$**
**Data collection**		
Beam line	IO3, Diamond	IO2, Diamond
Wavelength (Å)	0.97	0.98
Space group	$P2_12_12_1$	$P2_12_12_1$
Cell dimensions		
a, b, c (Å)	170.9, 177.1, 181.7	169.3, 176.0, 182.2
Resolution (Å)*	73.06 – 2.09 (2.15-2.09)	56.4 – 2.2 (2.26-2.2)
Total no of reflections	1575553	2166653
Unique reflections	316491	273854
Redundancy	4.9 (4.9)	7.9 (8.1)
Completeness (%9	99.2 (99.19	99.9 (100)
Mean $I/\sigma(I)$	20.1 (2.4)	13.0 (3.0)
R_{merge}	0.047 (0.659)	0.104 (0.638)
Solvent (%)	60	60
**Refinement**		
R_{work} / R_{free} (%)	22.3 / 24.3	23.7 / 27.6
r.m.s.d. Bonds(Å)/Angles(°)	0.009 / 1.36	0.008 / 1.245
No. atoms		
Protein	29089	28991
Solvent	976	654
NADP$^+$		564
B factors (Å^2)		
Protein	47.1	45.2
Water	43.2	42.3
NADP$^+$		40.3
Ramachandran plot		
Allowed/disallowed (%)	99.9 / 0.1	99.9 / 0.1
Molprobity score / percentile	1.67 / 94	1.75 / 95
PDB code	4B7C	4B7X

*Values in parenthesis refer to the highest resolution shell

Figure S1: Views of the refined electron density maps for the structures reported. A: Stereo view of the $2F_o$-F_c electron density map contoured at 1.8 σ for PA5259. B: Stereo view of parts of the $2F_o$-F_c Electron density map of PA2169 contoured at 1.7σ. C: View of the $2F_o$-F_c electron density map around the NADP cofactor in the PA4992-NADP complex contoured at 1.2σ. D: Stereo representation of the 2Fo-Fc electron density, contoured at 1σ, at the position of the conserved residue Asp70 of PA4485. E: Stereo representation of the active site of PA4098. Interactions between the NAD^+ and active site residues are represented and putative catalytic triad S133, Y146 and K159 is shown in green. The 2Fo-Fc electron density map for bound NAD^+ is contoured at 1σ. F: Part of the 2Fo-Fc electron density map, contoured at 1σ, showing one part of the β barrel of PA3770. G: Stereo representation of the apex of the funnel in the trimeric arrangement of PA1645. Residue Q105 alongside a bound sulphate ion are illustrated and appear to block the funnel. The 2Fo-Fc electron density map is contoured at 1σ. H: Stereo representation of the $NADP^+$ binding site in PA1648. The 2Fo-Fc electron density map is contoured at 1σ.

A:

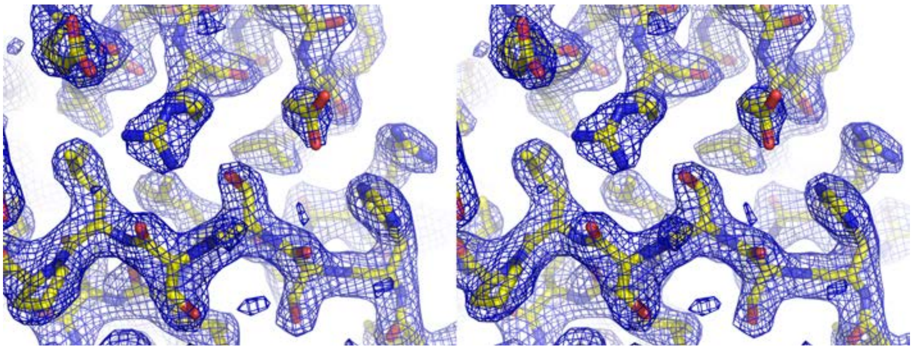

B:

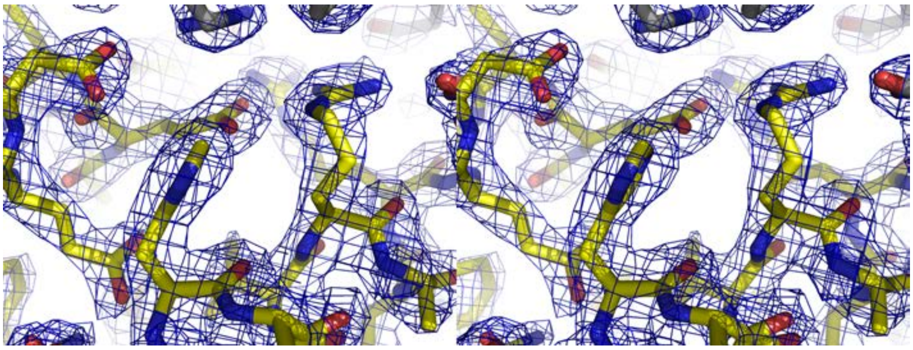

C:

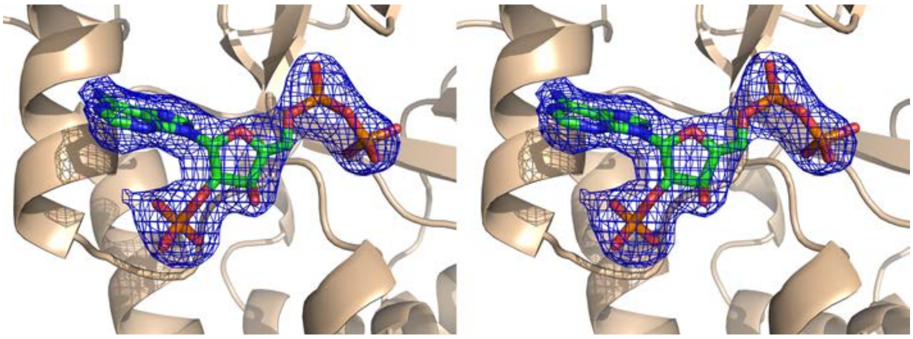

D:

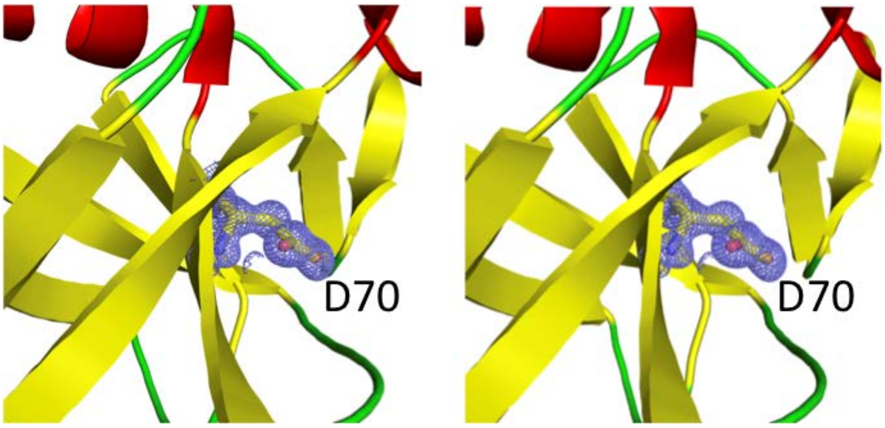

E:

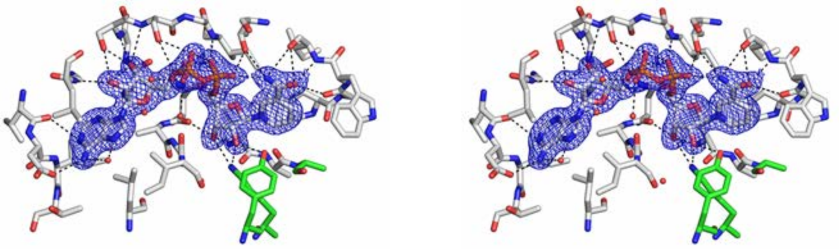

F:

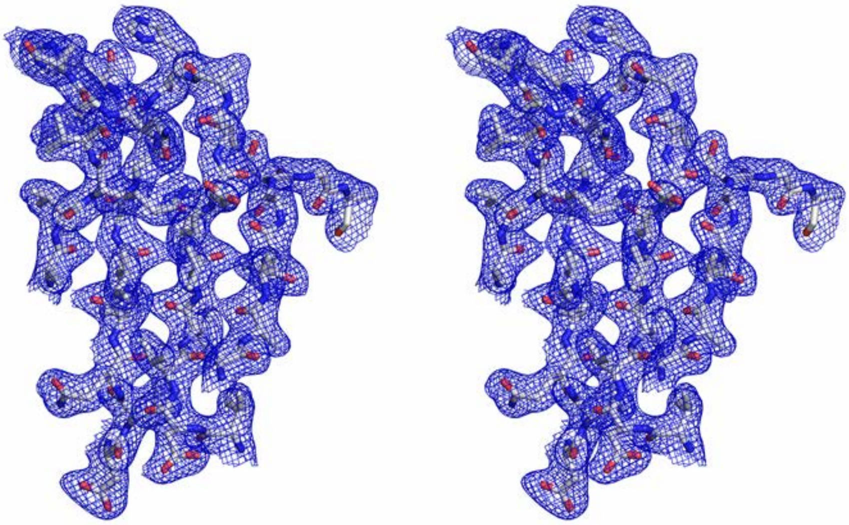

G:

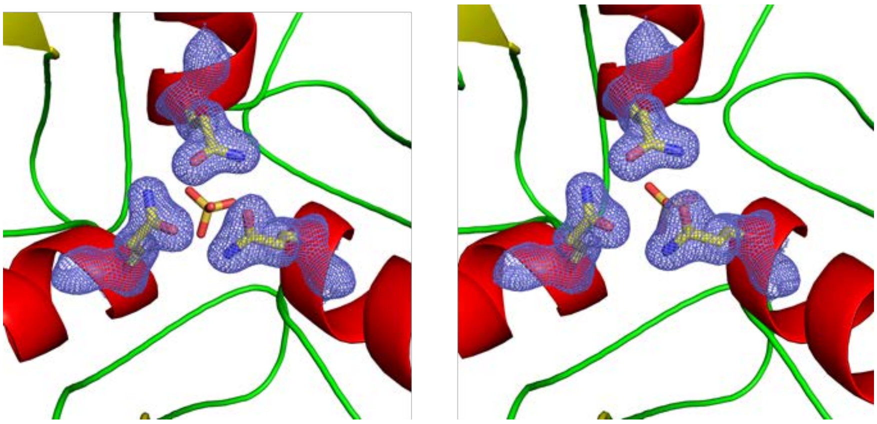

H:

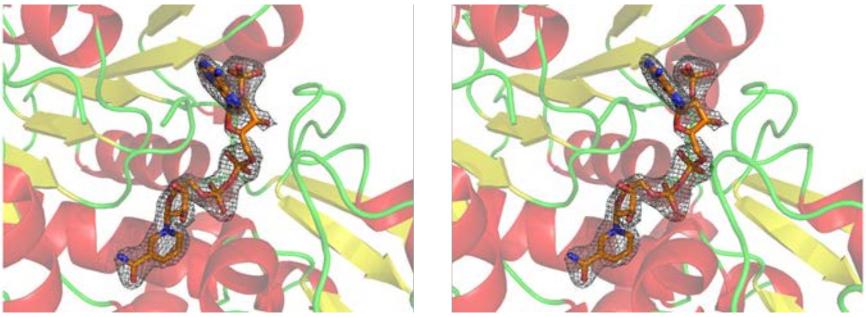

Supplement: Supplementary file 1 [file f-69-00025-sup1.pdf]
